# Possibilities for prevention reporting

**DOI:** 10.17886/RKI-GBE-2017-090

**Published:** 2017-08-30

**Authors:** Susanne Jordan, Gianni Varnaccia, Anne Starker

**Affiliations:** Robert Koch Institute, Department of Epidemiology and Health Monitoring, Berlin, Germany

## Abstract

The generally complex nature of interventions in disease prevention and health promotion pose particular challenges to establishing a system for prevention reporting. Comprehensive impact models and specific indicators that are capable of capturing risks as well as protective factors and also account for both behavioural and community factors should provide the basis. After health monitoring, we will also need to establish a system for the monitoring of interventions, policy and media.

## Need for prevention reporting

In recent years, the conditions for comprehensive and population-wide prevention reporting have improved. Research and practice in disease prevention and health promotion increasingly follow the Public Health Action Cycle approach, which means that activities are increasingly conducted within the four-step framework of assessment, policy development, assurance and evaluation. This method requires differentiated data to formulate prevention needs, identify prevention approaches and evaluate the impact of target-oriented prevention strategies. So far, in Germany, population-wide data on the implemented measures for disease prevention and health promotion (intervention reporting), their determinants and impacts is scant and based on few data sources [1]. The planned prevention report (to be produced by the National Prevention Conference every four years) should create an important incentive to improve data availability and is set to provide a basis to make the forms of and possibilites for prevention reporting in Germany clearer.

## Requirements for prevention reporting

For population-wide impacts, prevention measures will have to be harmonised to the greatest possible extent and need to intervene at multiple levels. To satisfy the complex demands that measures of disease prevention and health promotion need to meet, such multilevel interventions are considered a particularly promising approach. Measures should therefore be conceived with the aim of influencing risk and protective factors, accounting for local conditions in settings and improving the overall conditions (community, policy and environment) [2]. Accordingly, if such reporting wishes to describe municipal, regional or nationwide developments, all of these factors will also have to become part of prevention reporting.

## Impact models and monitoring for prevention reporting

**Figure 1:**
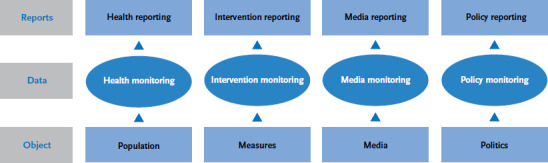
Types of monitoring and reporting Source: [1]

So-called impact models could provide the basis for a strategy to monitor disease prevention and health promotion measures. Impact models record the impact of health promotion and disease prevention measures by bringing together a variety of data on health outcomes, influencing factors and health determinants. They refer to different sources of data from surveys, routine data collection and evaluation activities. Impact models evidence causal relations between measures and (planned) effects and allow the assessment of all relevant influencing factors. Examples include the Result Model of Gesundheitsförderung Schweiz (Health Promotion Switzerland), which Switzerland applied to verify the national health goal Healthy body weight [3], or the framework model used by the World Health Organization to implement national strategies that promote healthy diets and physical activity [4]. Impact models make use of different types of monitoring depending on the kind of data they rely on and are then correspondingly referred to as health, intervention, media or policy monitoring (Figure 1).

## Indicators for prevention reporting

An impact model that forms the basis for prevention reporting will require adequate, verifiable and meaningful indicators which are available at the population level and can be surveyed at a reasonable expense. Correspondingly, this will require a diverse set of data sources: individual surveys, survey data acquired through health monitoring or official statistics and routine data, for example, from health insurance funds. However, concerning survey instruments and data availability, there is still significant need for further development. So far, for example, age-specific indicators and indicators capable of capturing protective factors are lacking.

## Conclusion for reporting on disease prevention and health promotion

In future, we should focus on using, and further developing, the available impact models and adapting them to reflect the goals of complex interventions in the fields of disease prevention and health promotion. Prevention reporting should include all areas of monitoring, i.e. intervention, policy and media monitoring in addition to health monitoring. To ensure this, we will need to promote the further development and establishment of indicators to operationalise resources and well-being as well as the use and the mapping of the structure of disease prevention and health promotion services.
